# Antecedents of malevolent creativity: a meta-analysis of personality and press predictors

**DOI:** 10.3389/fpsyg.2026.1846710

**Published:** 2026-07-22

**Authors:** Jiayi Zhu, Liyuan Liu, Zhesheng Ma, Xingnan Cui, Hua Zhang

**Affiliations:** 1 Faculty of Psychology, Southwest University, Chongqing, China; 2 Inner Mongolia Student Bullying Prevention Research Center, Tongliao, China; 3 Chengdu Caotang Primary School West Branch, Chengdu, China; 4 Center for Studies of Education and Psychology of Ethnic Minorities in Southwest China, Southwest University, Chongqing, China

**Keywords:** 4P model, malevolent creativity, meta-analysis, person, press

## Abstract

Malevolent creativity (MC) has received increasing attention in creativity research, yet findings on its antecedents have been heterogeneous. To address limitations of prior meta-analyses and incorporate a substantial number of recent studies, the present study provides an updated and methodologically rigorous meta-analysis of the links between MC and its antecedents. Guided by the 4P framework, we organized the literature into two broad categories—person and press—and examined each separately. A total of 362 effect sizes from 55 studies (*N* = 21,619) were analyzed using a three-level meta-analytic model. Negative person factors showed a significant positive association with MC, *r* = 0.33, whereas positive person factors showed a small negative association, *r* = −0.14. For press-related antecedents, negative press was positively associated with MC, *r* = 0.25, whereas positive press showed a small negative association, *r* = −0.13. Moderator analyses further indicated that these associations varied as a function of age, measurement approach, and indicator type, but were relatively stable across country of study and study quality. Implications for theory and practice and directions for future research are discussed.

**Systematic review registration:**

https://archive.org/details/osf-registrations-69v8x-v1; Identifier: doi: 10.17605/OSF.IO/69V8X.

## 1 Introduction

Creativity is often regarded as a positive force driving scientific invention, artistic production, and social progress. Yet creativity can also serve harmful ends. This darker side of creativity, commonly referred to as malevolent creativity (MC), refers to the intentional generation of novel ideas or behaviors aimed at harming others, often to gain an unfair advantage through manipulation, threat, or direct harm (Cropley et al., 2008). Its defining features are intentional harmfulness and originality, and manifestations range from interpersonal aggression and social manipulation to large-scale acts such as terrorism (Lee and Dow, 2011). Because such acts rely on novelty, they often evade existing rules and are difficult to anticipate or detect, amplifying their harmful consequences (Cropley et al., 2008; Cropley, 2010).

Although meta-analytic work on MC has begun to emerge, existing syntheses have focused primarily on outcomes and correlates of MC rather than systematically examining its antecedents. As a result, the relative contributions of person-level factors such as personality traits and press-level factors such as environmental stressors remain unclear, and the boundary conditions under which these effects are stronger or weaker have not been quantitatively examined. The present meta-analysis addresses this gap by providing the first comprehensive quantitative synthesis of the antecedents of MC within the 4P framework (Rhodes, 1961), focusing on person and press factors across 55 studies and 362 effect sizes.

### 1.1 Person and press factors

Most theoretical accounts of malevolent creativity have been drawn from the aggression and creativity literature. In the present work, we use the classic 4P framework as an organizing lens to reinterpret this field (Rhodes, 1961). The 4P framework conceptualizes creativity in terms of person, press, process, and product. This framework is particularly suitable for understanding malevolent creativity because the existing literature has consistently identified both dispositional and situational antecedents as its primary drivers, mapping directly onto the person and press components of the model. At the dispositional level, traits such as physical aggression, conscientiousness, and emotional intelligence have been linked to malevolent creativity, while at the situational level, environmental contexts such as perceived unfairness have been shown to elicit malevolent creative behavior (Cropley et al., 2008; Lee and Dow, 2011; Harris et al., 2013; for a review, see Storme et al., 2021). Moreover, consistent with prior meta-analytic work in creativity research, most empirical studies focus on one or two perspectives of the 4P model rather than all four simultaneously (Haase et al., 2025), suggesting that a focused examination of person and press factors is both theoretically defensible and methodologically appropriate. Here, we focus on person and press, as these two components capture the antecedent conditions most commonly examined in research on malevolent creativity. Person refers to individual characteristics, such as knowledge, experience, personality traits, attitudes, motivation, and persistence (Sternberg and Karami, 2022). Press refers to environmental forces that shape thinking and behavior, including ideological climates, social contexts, and external rewards or punishments (Simonton, 1994). While the remaining two components of the 4P model, product and process, are acknowledged as important aspects of malevolent creativity, they fall outside the scope of the present meta-analysis; their potential interactions with person and press factors represent a valuable direction for future research.

Viewed through this framework, research on malevolent creativity has largely developed along two broad lines. One line emphasizes person factors. Guided by models such as the General Model of Aggression and trait activation theory, this work suggests that aggression-prone individuals are more likely to think, interpret, and respond in malevolent ways (Lee and Dow, 2011; Harris and Reiter-Palmon, 2015). Consistent with this view, studies have linked malevolent creativity to traits such as narcissism, Machiavellianism, psychopathy, callousness, antagonism, and physical aggression (Perchtold-Stefan et al., 2021a; Batey et al., 2022; Silva et al., 2025). These traits may predispose individuals to generate harmful and novel solutions even in the absence of explicit situational prompts. For example, in decontextualized tasks such as the Alternate Uses Task (AUT), trait physical aggression has been found to significantly predict malevolent creativity performance (Lee and Dow, 2011). For person-related protective factors, evidence suggests that brighter personality traits, such as, conscientiousness, authenticity, honesty-humility, and agreeableness, are negatively associated with malevolent creativity (Gutworth et al., 2018; Shi et al., 2023; Xu et al., 2021). More broadly, positive traits may reduce malevolent intentions, strengthen ethical constraints, and promote prosocial forms of creativity, thereby serving as protective factors against malevolent creative behavior (Gao et al., 2025a).

A second line of research emphasizes press factors by using adverse or threatening situations to activate malevolent potential. Social information processing theory suggests that malevolent creativity is shaped by social context. By influencing what individuals perceive as acceptable, justified, or effective, environmental cues may channel the otherwise neutral process of creativity toward harmful ends (Gutworth et al., 2018). These studies argue that threat-related contexts and negative activating emotions, such as anger or fear, can facilitate malevolent idea generation (Perchtold-Stefan et al., 2024). Relevant situational cues include social exclusion, infectious disease threat, social threat, and unfairness (Baas et al., 2019; Ge et al., 2026; Wu et al., 2024; Zhang et al., 2024; Zhao et al., 2022a). Such contexts may increase anger, heighten aggressive tendencies, and promote desires for revenge or sabotage. They may also foster moral disengagement, particularly in provocative situations, making retaliatory aggression seem justified (Shi et al., 2023). Moreover, when individuals generate novel ways to punish a wrongdoer while avoiding detection or punishment, these ideas may increase feelings of efficacy and positive affect, thereby increasing the likelihood of endorsing or implementing malevolent solutions (Nguyen et al., 2025). Positive and supportive environmental factors, such as teacher support, emotional warmth, and family support, primarily arise from school and family contexts, are the two most important developmental settings in adolescence (Chen et al., 2025; Li et al., 2025b). High-quality family interactions and positive teacher–student relationships may foster prosocial values and moral guidance, thereby reducing the likelihood of malevolent creative behavior (Cui et al., 2024).

Therefore, building upon prior meta-analyses and existing literature in the field, a more rigorous approach encompassing a more comprehensive research corpus and more stringent methodological rigor is warranted. The 4P model is employed to categorize and synthesize the literature in the field of MC.

### 1.2 Possible moderator variables

Many factors may account for discrepancies in the direction and magnitude of associations between malevolent creativity (MC) and its antecedents. Based on the existing literature, we considered six candidate moderators in the present study: gender, age, country of study, study quality, MC measurement, and MC indicator.

#### 1.2.1 Gender

Research on gender differences in malevolent creativity has consistently demonstrated that males tend to generate more harmful or aggressive ideas than females across diverse methodological approaches. This pattern has been observed using both performance-based measures, such as the Alternate Uses Task (AUT; Dumas and Strickland, 2018), and self-report instruments, including the Malevolent Creativity Behavior Scale (MCBS; Xu et al., 2023; Zhao et al., 2022b). Neurophysiological evidence further corroborates these behavioral findings. Perchtold-Stefan et al. (2023a) reported gender-related differences in cortical activation and functional coupling within the EEG alpha band during malevolent creative ideation, suggesting distinct neural mechanisms underlying malevolent creativity in men and women. One plausible explanation for these observed differences is that males, particularly under conditions of stress, may exhibit heightened aggressive tendencies and a greater propensity to employ direct forms of aggression (e.g., physical aggression; Card et al., 2008; Lee and Dow, 2011). This interpretation aligns with prior meta-analytic evidence indicating that males, on average, demonstrate higher risk-taking propensity and greater willingness to engage with novel but uncertain ideas (Kim et al., 2024). These cognitive-behavioral tendencies may facilitate the generation of malevolent creative solutions.

#### 1.2.2 Age

Consistent with the well-established age-crime curve documented in criminological literature, developmental patterns of aggressive behavior exhibit a comparable lifespan progression, rising throughout early adolescence, peaking in late adolescence, and declining by the mid-twenties (Quetelet, 1831; Szabó et al., 2022). As a covert form of aggression, malevolent creativity may follow similar developmental trajectories. Indeed, emerging evidence suggests that the antecedents and mechanisms of malevolent creativity differ systematically across developmental stages. As a covert form of aggression, malevolent creativity may follow similar developmental trajectories, a possibility supported by a growing body of empirical evidence.

In adolescent samples, malevolent creativity appears to be primarily driven by proximal social risks and regulatory failures, including bullying victimization, anger, hostile attribution bias, deviant peer affiliation, and weaker self-control (Huang et al., 2026; Li et al., 2025a). These findings suggest that, during adolescence, malevolent creativity often emerges as a response to active social threat rather than as a stable dispositional trait. In contrast, studies of college students and emerging adults reveal a different pattern, in which earlier adversity, including childhood trauma, neglect, and adverse childhood experiences (ACEs), predicts malevolent creativity through dispositional pathways such as aggression, Dark Triad traits, reduced empathy, and lower social support (Li et al., 2022; Jia et al., 2020; Geng et al., 2024). Across both developmental stages, aggression-related and antisocial traits appear to serve as the most consistent mediating mechanisms linking developmental risk to malevolent creativity.

Given that the balance between aggression, antisocial personality, and self-regulation changes across development, and that the field examines these mechanisms differently in adolescents versus young adults, mean age was included as a moderator in the present meta-analysis to examine whether the strength of associations between antecedents and malevolent creativity varies as a function of sample age.

#### 1.2.3 Country of study

Cross-national variations in cultural values, religious orientations, and legal frameworks may systematically influence both the expression and evaluation of malevolent creativity. Prior meta-analytic work on creativity has identified sociocultural context as a significant moderator of creative outcomes (Abdulla Alabbasi et al., 2025; da Costa et al., 2015; Said-Metwaly et al., 2024). These effects may be particularly relevant for malevolent creativity, given that cultural dimensions such as collectivism vs. individualism and power distance may shape the extent to which individuals suppress or express malevolent impulses. In collectivist cultures, for instance, stronger norms of group harmony and interpersonal conformity may inhibit the open expression of malevolent creative behavior, whereas individualist cultures may afford greater tolerance for deviant or unconventional thinking (Triandis, 1989). Furthermore, cross-national differences in legal frameworks and moral norms may influence how malevolent creativity is perceived and reported across studies.

#### 1.2.4 Study quality

Study quality was included as a moderator because methodological variations across primary studies may systematically influence effect size estimates. Specifically, differences in sampling strategies, measurement reliability, control of confounds, and study design rigor have been shown to produce heterogeneity in meta-analytic findings across various psychological domains (Chandler et al., 2019). In the context of malevolent creativity research, which encompasses both experimental and correlational designs with varying levels of methodological rigor, it is plausible that higher-quality studies may yield more conservative or more precise effect size estimates than lower-quality ones. Including study quality as a moderator therefore allows us to assess the robustness of the observed associations and to determine whether effect sizes are inflated by methodologically weaker studies. To operationalize study quality, we used the QualSyst tool (Kmet et al., 2004), which is appropriate for both experimental and correlational study designs (Riddell et al., 2025).

#### 1.2.5 Malevolent creativity measurement

Malevolent creativity appears to vary systematically depending on the method of measurement, and this variation may reflect genuine differences in the constructs being assessed rather than mere methodological noise. The two most widely used instruments in the literature, the Malevolent Creativity Behavior Scale (MCBS; Hao et al., 2016) and the Malevolent Creativity Task (MCT; Lee and Dow, 2011), differ fundamentally in their design and what they capture. The MCBS is a self-report questionnaire that assesses the frequency and tendency of malevolent creative behaviors in everyday life, and has been shown to correlate significantly with stable personality traits such as aggression, openness, and extraversion. As such, it functions primarily as a trait-level measure of dispositional tendencies toward malevolent behavior. In contrast, the MCT is an acute experimental task that places participants in a conflict-laden situation and requires them to generate novel malevolent ideas under conditions of tension and opposition, thereby capturing situationally elicited malevolent ideation rather than stable behavioral tendencies. These fundamental differences in operationalization suggest that MCBS and MCT may be differentially sensitive to person-level vs. press-level antecedents of malevolent creativity. Specifically, trait-level measures such as the MCBS may be more strongly predicted by stable personality characteristics, whereas situational measures such as the MCT may be more sensitive to environmental press. Given these considerations, MC measurement tool was included as a moderator in the present meta-analysis to examine whether effect sizes differ systematically as a function of the instrument used.

#### 1.2.6 Malevolent creativity indicator

The specific indicators used to assess malevolent creativity performance may moderate the relationship between antecedent variables and malevolent creativity. Most studies report commonly used creativity indicators such as fluency, originality, and flexibility; some additionally assess malevolence, usefulness, or harmfulness (Gutworth et al., 2018; Perchtold-Stefan et al., 2022b). Although these indicators are highly correlated, they reflect distinct cognitive processes and may therefore relate differently to antecedent variables (Gao et al., 2022a). Specifically, fluency reflects the quantity of malevolent ideas generated and may be more sensitive to situational activation such as environmental press, which broadly increases idea generation. Originality, by contrast, reflects the uniqueness and novelty of malevolent solutions, which may be more strongly tied to stable cognitive traits such as openness to experience or divergent thinking ability. Meanwhile, indicators specific to malevolent creativity, including malevolence, harmfulness, and usefulness, capture the degree to which generated ideas are genuinely harmful or practically applicable. Given these theoretical distinctions, MC indicator was included as a moderator in the present meta-analysis to examine whether the strength of associations between antecedents and malevolent creativity varies as a function of the specific creativity dimension assessed.

### 1.3 The present study

Empirical studies on malevolent creativity (MC) and its antecedents have produced mixed results, with some reporting positive and others negative associations with person factors. Prior meta-analytic work offered preliminary insights but was limited by methodological issues and incomplete coverage of the literature and potential moderators (Zhou et al., 2024). To address these gaps, we included studies examining both press and person factors, guided by the 4P framework for classification (Rhodes, 1961). Person factors were further divided into cognitive factors, personality traits, and state-trait hybrids, with a primary focus on personality traits (Buss and Finn, 1987; Endler, 2000; Gfrörer et al., 2022). This allowed us to examine whether MC is more strongly associated with press or person factors. Using modern, robust meta-analytic techniques, we tested moderators to explain variability in effect sizes. Our analysis addresses two questions: (1) How strong are the associations between MC and its antecedents? (2) Are MC outcomes more closely linked to person factors or press factors?

## 2 Method and materials

### 2.1 Literature search and inclusion criteria

First, four electronic databases (Web of Science, ProQuest, ScienceDirect, and APA PsycInfo) were searched for articles published up to December 2025. Titles and abstracts were searched using the key term “malevolent creativity.” Second, a backward search was conducted by examining references cited in relevant articles, including the prior meta-analysis by Zhou et al. (2024). The titles and abstracts of articles retrieved through the search strategy were screened using Rayyan (Ouzzani et al., 2016). The first and second authors independently reviewed all records and excluded those that were clearly irrelevant to the present meta-analysis. Articles deemed potentially relevant were subjected to full-text assessment. To be included in the meta-analysis, studies had to meet the following criteria (Pigott and Polanin, 2020): (a) published in English with a full-text version accessible; (b) explicitly examine the association between malevolent creativity and one or more of its antecedents (studies that assessed malevolent creativity solely via creative choice tasks were excluded, as were studies that measured malevolent creativity without assessing any putative antecedent variables); and (c) provide sufficient quantitative information to allow for the calculation of effect sizes. In cases of disagreement between the two reviewers regarding eligibility, the corresponding author independently evaluated the article's relevance and made the final inclusion decision. This meta-analysis was preregistered on the Open Science Framework (OSF; https://osf.io/gs89v?view_only=cc9524754fa640cebf665a3f648a0bf0). All data and analysis code are publicly available at the same repository.

### 2.2 Data coding

For each eligible article, the publication year, authors, sample size, antecedent variables, and reported effect size were recorded. The following moderator variables were coded: First, gender was coded as a continuous variable reflecting the proportion of female participants in each sample. Second, population country was coded as a categorical variable with the following levels: China, United States, Austria, Brazil, United Kingdom, Italy, Pakistan, Portugal, the Netherlands, and Other (for studies that did not specify the country of data collection). Third, participant age was coded as a continuous variable using the reported mean and standard deviation. Fourth, PE type (person–environment interaction) was coded as a categorical variable with two levels: person and environment (da Costa et al., 2015). Fifth, variable valence was coded as a categorical variable indicating whether the antecedent was conceptualized as positive, negative, or other. Sixth, malevolent creativity indicator was coded as a categorical variable based on the specific facet measured, including: total score, malevolence, originality, fluency, harmfulness, flexibility, usefulness, hurting people, lying, and playing tricks. Seventh, malevolent creativity measurement was coded as a categorical variable based on the instrument used: Malevolent Creativity Behavior Scale (MCBS) (Hao et al., 2016), Malevolent Creativity Test (MCT) (Lee and Dow, 2011; Perchtold-Stefan et al., 2022b), Malevolent Divergent Thinking task (MDT) (Batey et al., 2022), Alternate Uses Task adapted for malevolence (AUT) (Harris et al., 2013), or Other. In the coding process, we adopted the same strategy as Said-Metwaly et al. (2024). When a study reported both total-scale and subscale correlation coefficients between the MCBS and antecedent variables (e.g., hurting people, lying, and playing tricks), only the subscale correlations were retained to avoid redundancy.

### 2.3 Analyses

The effect size metric selected for the present meta-analysis was Pearson's *r*. To address violations of normality in the sampling distribution of correlation coefficients, all *r* values were transformed to Fisher's *z* prior to analysis. Following aggregation, results were back-transformed to the original *r* metric to facilitate interpretation (Card, 2015). A random-effects model was adopted, as the included primary studies were regarded as a random sample drawn from a larger population of possible studies (Pigott and Polanin, 2020). Conventional meta-analytic approaches assume independence among effect sizes; however, when a single study reports multiple effect sizes, traditional methods often retain only one estimate, which can reduce statistical power and bias parameter estimates (Cheung, 2014). To address this limitation, a three-level random-effects meta-analytic model was employed. This approach explicitly accounts for the hierarchical structure of the data by modeling three sources of variance: (a) sampling variance (Level 1), (b) within-study variance across dependent effect sizes (Level 2), and (c) between-study variance (Level 3). By doing so, the model retains all available effect sizes without requiring aggregation or selection, thereby preserving statistical power and making full use of the information provided by each study (Assink et al., 2015; Said-Metwaly et al., 2024). Following Viechtbauer and Cheung (2010), we used studentized residuals and Cook's distances to identify outlying and influential studies. We then conducted leave-one-out sensitivity analyses by removing one effect size at a time and re-estimating the pooled effect size. All analyses were conducted in R (Version 4.4.1) using the metafor package (Viechtbauer, 2010), following the implementation guidelines provided by Assink and Wibbelink (2016) for both overall effect estimation and moderator analyses.

## 3 Results

### 3.1 Overview of included articles

**Figure 1:**
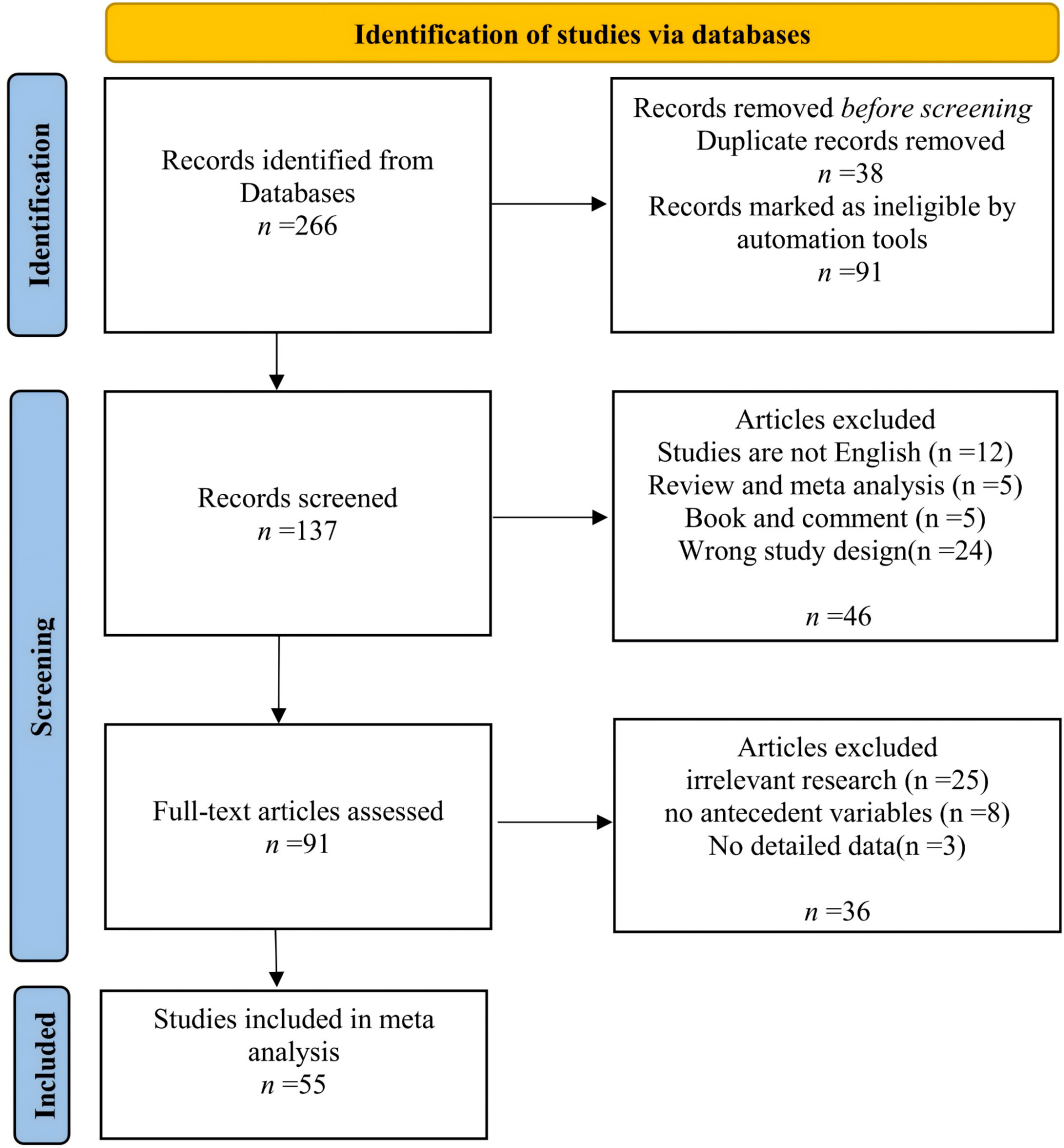
Identification of studies via databases.

A flowchart of the search process is presented in Figure 1. Our search of databases, supplemented by backward searches of prior reviews and meta-analyses, identified 266 records. After applying the inclusion and exclusion criteria, 55 studies were retained, yielding 362 effect sizes and a total sample of 21,619 participants. Sample sizes ranged from 50 to 1,501, and the proportion of females ranged from 0% to 100%. Most samples were drawn from China (49.72%).

### 3.2 Overall analysis

For press-related antecedents, negative press showed a significant positive association with malevolent creativity, *r* = 0.258, 95% CI [0.206, 0.311], 95% PI [−0.027, 0.544], *k* = 80, *p* < 0.001. The prediction interval crossed zero, suggesting that while the mean effect is positive and reliable, considerable variability exists across studies and the effect in any new individual study may range from negligible to moderately large. Heterogeneity was significant, *Q*_(79)_ = 484.40, *p* < 0.001, with 40.0% of the total variance at the between-study level and 60.0% at the within-study level. In contrast, positive press was not significantly associated with malevolent creativity, *r* = −0.132, 95% CI [-0.289, 0.026], 95% PI [-0.545, 0.282], *k* = 9, *p* =0.102. The wide prediction interval further reflects substantial uncertainty, consistent with the significant heterogeneity observed, *Q*_(8)_ = 144.96, *p* < 0.001, with 81.6% of the total variance at the between-study level and 18.4% at the within-study level.

For person-related antecedents, negative person factors showed a significant positive association with malevolent creativity, *r* =0.342, 95% CI [0.219, 0.465], 95% PI [−0.216, 0.900], *k* = 107, *p* < 0.001. Although the mean effect is significant and moderate in magnitude, the prediction interval was notably wide and crossed zero, indicating that the effect of negative personality traits on malevolent creativity varies considerably across studies and contexts. Heterogeneity was substantial, *Q*_(106)_ = 2520.43, *p* < 0.001, with 77.92% of the total variance at the between-study level and 22.08% at the within-study level. By contrast, positive person factors showed a small but significant negative association with malevolent creativity, *r* = −0.142, 95% CI [−0.268, −0.015], 95% PI [−0.630, 0.347], *k* = 38, *p* =0.028. The prediction interval again crossed zero, suggesting meaningful variability across studies. Heterogeneity was also significant, *Q*_(37)_ = 511.54, *p* < 0.001, with 91.38% of the total variance at the between-study level and 8.62% at the within-study level. Because the number of studies in the positive category was limited and this category was less central to our research question, we did not include it in subsequent analyses.

To formally address the main research question, whether personality-based antecedents exert a stronger influence on malevolent creativity than environmental antecedents, we fitted a combined three-level meta-analytic model with group membership as a moderator (negative person = 1, negative press = 0). The results revealed that the effect of negative person factors was significantly greater than that of negative press, *B* = 0.168, *SE* = 0.043, *z* = 3.938, *p* < 0.001, 95% CI [0.085, 0.252], *QM*_(1)_ = 15.511, *p* < 0.001. This indicates that, while both negative person factors (*r* =0.342) and negative press (*r* = 0.258) were significantly associated with malevolent creativity, personality-based antecedents exerted a significantly stronger influence than environmental antecedents, thus formally confirming the main research question of the present study.

### 3.3 Sensitivity analyses

Sensitivity analyses indicated that the findings were robust across all four sets of effects. For negative press, four effect sizes had studentized residuals exceeding the conventional cutoff of 1.96, although all Cook's distance values were below 0.45; the pooled effect size ranged from *r* = 0.246 to *r* = 0.267 across leave-one-out analyses. For positive press, all effect sizes fell within the acceptable range for both studentized residuals and Cook's distances, and the pooled effect size ranged from *r* = −0.178 to *r* = −0.084. For negative person factors, two effect sizes had studentized residuals greater than 1.96, but all Cook's distance values remained below 0.45; the pooled effect size ranged from *r* = 0.317 to *r* = 0.351. For positive person factors, one effect size exceeded the 1.96 cutoff for studentized residuals, whereas all Cook's distance values were below 0.45; the pooled effect size ranged from *r* = −0.175 to *r* = −0.112. Together, these results suggest that no single effect size exerted undue influence on the overall estimates.

### 3.4 Publication bias

**Figure 2:**
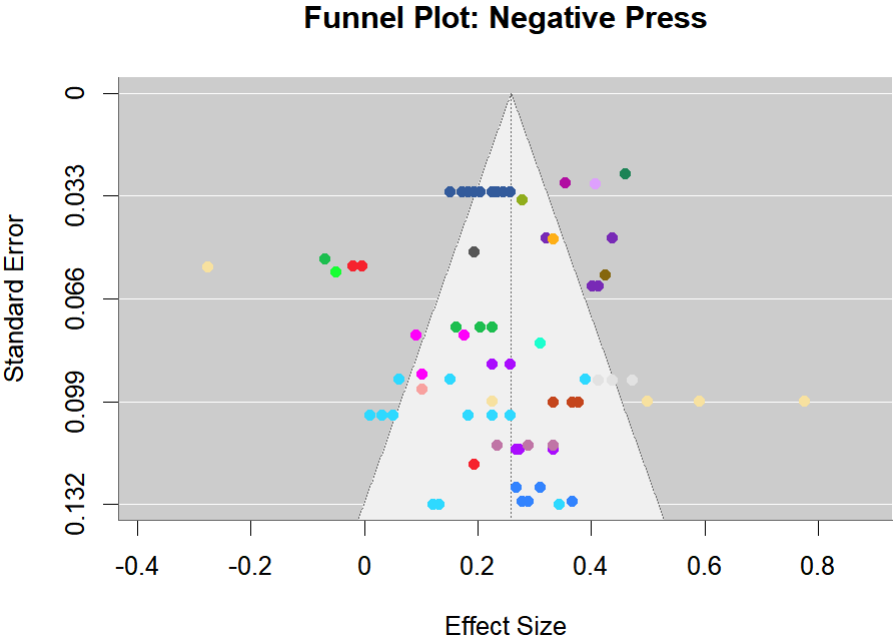
Funnel plot: negative press.

**Figure 3:**
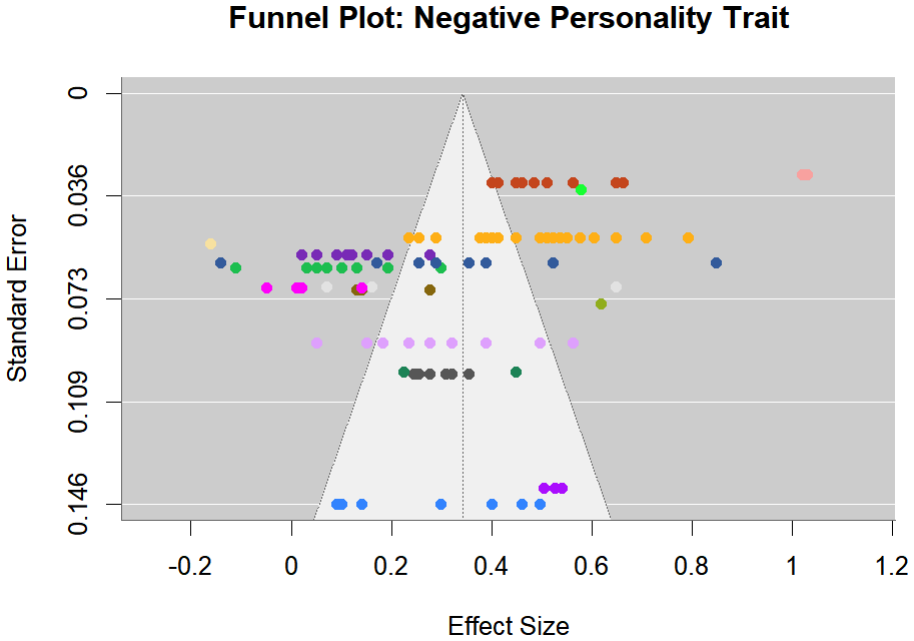
Funnel plot: negative personality trait.

Publication bias was assessed using Egger's regression test to account for dependent effect sizes (Fernández-Castilla et al., 2021). For the negative press subgroup, the slope coefficient was not significant, *t*_(11.0)_ = 1.20, *p* = 0.256 (Figure 2). Similarly, no significant funnel plot asymmetry was detected for the negative personality trait subgroup, *t*_(4.23)_ = −0.68, *p* = 0.530 (Figure 3). Visual inspection of funnel plots confirmed approximate symmetry for both subgroups. Collectively, these results suggest minimal evidence of publication bias.

### 3.5 Moderator analyses

We examined a range of potential moderators of the associations between negative person and press factors and malevolent creativity. Results are presented in Table 1 to Table 4, which can be found in the supplementary materials. Subgroups based on a single study (*k* = 1) were retained for descriptive purposes but should be interpreted with caution, as heterogeneity cannot be estimated and confidence intervals are necessarily wide. For example, in Table 4, the Brazilian subgroup yielded CI = −0.116, 1.128, Pakistan yielded CI = −0.051, 1.288, Portuguese yielded CI = −0.128, 1.174, and UK yielded CI = −0.500, 0.749. In Table 1, Austria yielded CI = 0.046, 0.523, Pakistan yielded CI = 0.135, 0.712, and the Netherlands yielded CI = −0.224, 0.424.

**Table 1:** Categorical moderator analyses for the association between negative press factors and malevolent creativity.

Categorical moderator	*m*	*k*	*I^2^*	τ	*Q*	*r*	SE	95% CI	*p*
**Country**			63.3	0.082	7.661				0.105
American	3	6				0.090	0.076	−0.059, 0.238	0.236
Austria	1	3				0.278	0.122	0.046, 0.523	0.019
China	16	69				0.270	0.028	0.222, 0.332	< 0.001
Pakistan	1	1				0.402	0.147	0.135, 0.712	0.004
the Netherlands	1	1				0.100	0.165	−0.224, 0.424	0.544
**MC type**			87.8	0.281	49.395				< 0.001
MCBS	12	26				0.058	0.068	−0.075, 0.190	0.394
MCT	12	54				0.425	0.067	0.321, 0.583	< 0.001
**MC dimension**			87.8	0.098	7.207				0.514
hurting people	1	4				0.200	0.112	−0.019, 0.425	0.074
Lying	1	4				0.222	0.112	0.004, 0.449	0.046
Malevolence	7	9				0.339	0.058	0.237, 0.466	< 0.001
MC harmfulness	3	7				0.145	0.071	0.007, 0.285	0.040
MC flexibility	1	2				0.294	0.118	0.066, 0.537	0.012
MC fluency	7	13				0.253	0.051	0.155, 0.361	< 0.001
MC originality	11	18				0.286	0.044	0.204, 0.382	< 0.001
Playing tricks	1	4				0.203	0.112	−0.017, 0.428	0.0070
total score	15	19				0.241	0.039	0.170, 0.322	< 0.001

*m* = number of studies; *k* = number of effect sizes; *I*^2^ = proportion of total variance attributable to between-study heterogeneity; τ = standard deviation of true effects; *Q* = heterogeneity statistic; *r* = mean effect size (Fisher's *z* back-transformed); *SE* = standard error; *CI* = confidence interval; *MC* = malevolent creativity; *MCBS* = Malevolent Creativity Behavior Scale; *MCT* = Malevolent Creativity Test. Subgroups with *k* = 1 should be interpreted with caution, as heterogeneity cannot be estimated and confidence intervals are necessarily wide.

**Table 2:** Continuous moderator analyses for the association between negative press factors and malevolent creativity.

Continuous moderator	*m*	*k*	*I^2^*	τ	*Statistics*	*B*	*SE*	*p*
Publication year	22	80	87.5	0.093	*t*_(78)_ = 0.620	0.009	0.015	0.535
Female ratio	22	80	87.0	0.084	*t*_(78)_ = −0.736	−0.001	0.002	0.462
Age	19	66	88.9	0.081	*t*_(64)_ = −1.665	−0.009	0.005	0.096
Age SD	18	65	88.9	0.083	*t*_(63)_ = −1.575	−0.018	0.012	0.115
Quality	22	80	87.0	0.090	*t*_(78)_ = 0.329	0.030	0.091	0.742

*M* = number of studies; *k* = number of effect sizes; *I*^2^ = proportion of total variance attributable to between-study heterogeneity; τ = standard deviation of true effects; *B* = unstandardized regression coefficient; *SE* = standard error.

**Table 3:** Continuous moderator analyses for the association between negative person factors and malevolent creativity.

Continuous moderator	*m*	*k*	*I^2^*	τ	*Statistics*	*B*	SE *B*	*p*
Publication year	17	107	84.6	0.222	*t*_(105)_ = 1.886	0.032	0.017	0.059
Female ratio	16	99	88.5	0.270	*t*_(97)_ = −0.606	−0.002	0.004	0.544
Age	13	79	88.3	0.280	*t*_(77)_ = 0.776	0.010	0.013	0.438
Age SD	11	72	89.0	0.289	*t*_(70)_ = 0.295	0.007	0.023	0.768
Quality	17	107	84.8	0.223	*t*_(105)_ = 1.797	0.198	0.110	0.072

*M* = number of studies; *k* = number of effect sizes; *I*^2^ = proportion of total variance attributable to between-study heterogeneity; τ = standard deviation of true effects; *B* = unstandardized regression coefficient; *SE* = standard error.

**Table 4:** Categorical moderator analyses for the association between negative person factors and malevolent creativity.

Categorical moderator	*K*	*m*	*I^2^*	τ	*Q*	*r*	*SE*	95% CI	*p*
**Country**			89.5	0.316	3.198				0.784
American	2	16				0.066	0.226	−0.376, 0.510	0.768
Austria	2	8				0.308	0.231	−0.139, 0.767	0.175
Brazilian	1	24				0.467	0.317	−0.116, 1.128	0.111
China	6	27				0.350	0.133	0.102, 0.623	0.006
Pakistan	1	1				0.567	0.342	−0.051, 1.288	0.070
Portuguese	1	3				0.484	0.332	−0.128, 1.174	0.115
UK	1	10				0.123	0.319	−0.500, 0.749	0.697
**MC_type**			94.9	0.205	11.707				0.003
MCBS	14	74				0.400	0.059	0.297, 0.490	< 0.001
MCT	5	23				0.167	0.080	0.012, 0.315	0.035
MDT	1	10				0.123	0.209	−0.278, 0.490	0.553
**MC dimension**	17	107	84.4	0.222	19.129				0.014
hurting people	6	22				0.402	0.086	0.252, 0.533	< 0.001
Lying	6	22				0.315	0.086	0.155, 0.462	< 0.001
Malevolence	3	13				0.226	0.111	0.013, 0.426	0.038
MC harmfulness	1	1				−0.028	0.180	−0.363, 0.313	0.878
MC fluency	2	6				0.296	0.100	0.108, 0.469	0.002
MC originality	4	5				0.157	0.098	−0.034, 0.341	0.107
Playing tricks	5	18				0.333	0.088	0.171, 0.481	< 0.001
total score	10	18				0.393	0.075	0.263, 0.512	< 0.001
Usefulness	1	2				0.083	0.146	−0.201, 0.360	0.572

*M* = number of studies; *k* = number of effect sizes; *I*^2^ = proportion of total variance attributable to between-study heterogeneity; τ = standard deviation of true effects; *Q* = heterogeneity statistic; *r* = mean effect size (Fisher's *z* back-transformed); *SE* = standard error; *CI* = confidence interval; *MC* = malevolent creativity; *MCBS* = Malevolent Creativity Behavior Scale; *MCT* = Malevolent Creativity Test. Subgroups with *k* = 1 should be interpreted with caution, as heterogeneity cannot be estimated and confidence intervals are necessarily wide.

For person-related factors, country of study, *QM*_(4)_ = 7.66, *p* = 0.105, publication year, *B* = 0.032, *p* = 0.059, female ratio, *B* = −0.002, *p* = 0.054, age standard deviation, *B* = 0.007, *p* = 0.077, and study quality, *B* = 0.198, *p* = 0.072, were not significant. Mean age was not a significant moderator, *B* = 0.01, *SE* = 0.013, *p* =0.438, *k* = 79, as 28 effect sizes were excluded due to missing age data. MC indicator, *QM*_(8)_ = 19.13, *p* = 0.014, and MC measurement, *QM*_(2)_ = 11.71, *p* = 0.003, also significantly moderated effect sizes. The largest effect was observed for Hurting People (*r* =0.43), whereas MC harmfulness showed the smallest effect (*r* = −0.03). *Post hoc* comparisons showed that studies using the Malevolent Creativity Behavior Scale (MCBS; *r* = 0.40) yielded larger effects than those using the Malevolent Creativity Task (MCT; *r* = 0.17), *B* = −0.25, *p* = 0.001. Effects for the Malevolent Divergent Thinking Task (MDT; *r* = 0.12) did not differ significantly from those for MCBS, *p* = 0.174. For negative press factors, publication year, *B* = 0.009, *p* = 0.535, female ratio, *B* = −0.001, *p* =0.462, mean age, *B* = −0.009, *p* = 0.096, age standard deviation, *B* = −0.018, *p* = 0.115, study quality, *B* = 0.030, *p* = 0.742, country of study, *QM*_(4)_ = 7.66, *p* = 0.105, and MC indicator, *QM*_(8)_ = 7.21, *p* = 0.514, were not significant. MC measurement significantly moderated effect sizes, *QM*_(1)_ = 49.40, *p* < 0.001. Studies using the Malevolent Creativity Task (MCT; *r* = 0.42) yielded larger effects than those using the Malevolent Creativity Behavior Scale (MCBS; *r* = 0.06), *B* = 0.39, *p* < 0.001.

To formally examine whether the divergence between MCBS and MCT reflected genuine instrument-level differences rather than confounding by sample characteristics, we conducted controlled multilevel meta-analytic models in which MC type was entered as a predictor alongside all other moderator variables examined in the present study, including publication year, mean age, age SD, female ratio, study quality, country of study, and MC indicator. These moderators were selected because they represent the full set of variables systematically examined in the present meta-analysis and therefore constitute the most comprehensive set of available controls. By simultaneously including all examined moderators, this approach allows us to isolate the unique contribution of instrument type to the observed effect size differences, independent of other study-level characteristics. For negative press factors, after controlling for all moderator variables, the difference between MCT and MCBS remained highly significant, *B* = 0.647, *SE* = 0.210, *z* = 3.083, *p* = 0.002, *k* = 65. For negative person factors, the difference remained marginally significant after controlling for all moderator variables, *B* = −0.442, *SE* = 0.225, *z* = −1.959, *p* = 0.050, *k* = 63. These results indicate that the divergence between MCBS and MCT cannot be attributed to differences in sample characteristics or other study-level variables. It should be noted that the full controlled models were based on reduced samples due to missing data on one or more moderator variables, and some country-level categories were dropped due to redundancy; these constraints should be considered when interpreting the controlled analyses. Furthermore, for negative press factors, MCBS studies had a substantially larger mean sample size (*N* = 771) than MCT studies (*N* = 118), despite being based on an equal number of independent studies (*m* = 12 each), suggesting that the null finding for MCBS cannot be attributed to insufficient statistical power. For negative person factors, the imbalance between MCBS (*m* = 14 studies) and MCT (*m* = 5 studies) is more pronounced and represents a limitation that should be considered when interpreting instrument-type comparisons in this category.

## 4 Discussion

### 4.1 Overall discussion

The present findings can be integrated into a coherent influence pathway grounded in the distinction between proactive and reactive aggression (Miller and Lynam, 2006). We propose that person-level and press-level antecedents contribute to malevolent creativity through two functionally distinct but potentially interacting routes. Person-related antecedents, particularly negative personality traits such as Dark Triad characteristics, physical aggression, and moral disengagement, operate primarily through a proactive pathway. Consistent with trait activation theory and the General Model of Aggression (Lee and Dow, 2011; Harris and Reiter-Palmon, 2015), individuals high in these traits may chronically maintain hostile cognitive schemas, selectively attend to threatening social cues, and sustain a reduced threshold for harmful ideation even in the absence of explicit situational provocation (Crick and Dodge, 1996). This proactive route reflects a stable, dispositionally driven form of malevolent creativity that is relatively independent of situational context. In contrast, press-related antecedents operate primarily through a reactive pathway. Adverse environmental conditions such as social exclusion, perceived unfairness, and provocation elicit negative activating emotions, particularly anger, which broadens the search for retaliatory responses while temporarily reducing moral constraints on harmful ideation (Berkowitz, 1990; Bandura, 2017). This reactive route reflects a situationally triggered form of malevolent creativity that is more context-dependent and emotionally driven.

Importantly, these two pathways are not independent. Individuals high in dark personality traits may be more reactive to negative environmental press, such that the combination of dispositional risk and situational provocation produces compounding effects that exceed those of either factor alone. This interaction represents the highest-risk configuration and warrants targeted attention in future research.

The present study offers an updated and more differentiated account of the antecedents of malevolent creativity. Consistent with Zhou's prior meta-analytic findings, negative person factors showed a significant positive association with malevolent creativity, *r* = 0.33. This pattern is in line with earlier evidence identifying moral disengagement, aggression, Dark Triad traits, and hostility as important correlates of malevolent creativity. Extending prior work, we also found that positive person factors showed a small negative association with malevolent creativity, *r* = −0.14. For press-related antecedents, negative press was positively associated with malevolent creativity, *r* = 0.25, whereas positive press showed a small negative association, *r* = −0.13. The fact that all prediction intervals crossed zero, despite significant mean effects, indicates substantial cross-study variability in the strength and direction of these associations. This pattern suggests that the relationships between antecedents and malevolent creativity are highly context-dependent, and that the moderators examined in the present study, while statistically significant, capture only a portion of this variability.

This residual heterogeneity likely reflects several sources of unaccounted variance. First, the included studies varied considerably in sample characteristics, cultural contexts, and methodological approaches, all of which may moderate the strength of antecedent-MC associations in ways that were not captured by the moderators examined here. Second, the moderators included in the present study were constrained by the variables reported in primary studies, which may not have captured the most theoretically relevant boundary conditions. Third, person and press factors may interact in ways that individual moderator analyses cannot detect, such that the effect of a given personality trait depends on the specific environmental context in which it is expressed. These considerations highlight the need for primary studies that systematically manipulate or measure both person and press factors simultaneously, allowing for the examination of interactive effects.

Moderator analyses further indicated that mean age was not a significant moderator of person-related effects, *B* = 0.010, *SE* = 0.013, *p* = 0.438, *k* = 79. Although an earlier version of this manuscript reported a significant age effect, this was identified as an error arising from an earlier version of the analysis. The corrected result indicates that age did not significantly moderate the association between negative person factors and malevolent creativity, though the direction of the effect was consistent with developmental accounts suggesting that age-related intellectual development may support the generation of more refined and deliberate novel ideas by expanding knowledge and increasing cognitive flexibility (Batey et al., 2022; Szabó et al., 2022). MC measurement significantly moderated effect sizes in both the negative press and negative person categories.

A notable divergence was observed in the effects of MCBS and MCT across antecedent types. For negative press factors, the MCBS yielded a non-significant effect (*r* = 0.06, *k* = 26), whereas the MCT produced the largest effect in the entire study (*r* =0.42, *k* = 54). This complete reversal across antecedent type is consistent with the proposed proactive-reactive framework. Specifically, the MCBS, as a self-report measure of everyday malevolent behavior frequency, is better suited to capture proactive malevolent creativity rooted in stable dispositional tendencies, and therefore shows stronger associations with person-level antecedents. The MCT, as an acute experimental task that places participants in a conflict-laden situation requiring the generation of novel harmful ideas under provocation, is structurally designed to elicit reactive malevolent creativity, and therefore shows stronger sensitivity to environmental press. This pattern may reflect fundamental differences in what these two instruments measure. The MCBS assesses the frequency and tendency of malevolent creative behaviors in everyday life, and has been shown to correlate significantly with stable personality traits such as aggression, openness, and extraversion (Hao et al., 2016). Consistent with the reactive aggression pathway, the MCT's situational design may render it particularly sensitive to environmental press, as the task itself simulates the kind of provocative context that negative press represents (Perchtold-Stefan et al., 2022b). It should be noted, however, that the difference in the number of effect sizes between MCBS (*k* = 26) and MCT (*k* = 54) for negative press may have contributed to the observed discrepancy in statistical significance, and this possibility should be considered when interpreting these findings. The robustness of this instrument-level difference was further confirmed by controlled multilevel models that accounted for all available moderator variables. The divergence between the two instruments remained significant for negative press (*B* = 0.647, *p* = 0.002) and marginally significant for negative person factors (*B* = −0.442, *p* = 0.050) after controlling for publication year, age, female ratio, study quality, country, and MC indicator, suggesting that the observed pattern reflects genuine construct-level differences rather than sampling artifacts. It should also be acknowledged that the disparity in study numbers between MCBS and MCT, particularly for negative person factors where MCBS drew on 14 studies compared with only 5 for MCT, limits the precision of effect size estimates for MCT and reduces the statistical power available to detect instrument-type differences in this category. Future research should prioritize a more balanced representation of MCBS and MCT studies to enable more definitive conclusions about instrument-level differences in sensitivity to person-level antecedents. It is also noteworthy that the residual heterogeneity associated with MC type as a moderator differed substantially across antecedent categories. For negative person factors, the MC type moderator yielded a residual *I*^2^ of 94.9%, compared with 87.8% for negative press factors. This pattern suggests that the divergence between MCBS and MCT is not only more pronounced in terms of effect size direction for negative press, but that the negative person category also shows greater between-study variability in MC type effects. One possible explanation is that the relationship between dark personality traits and malevolent creativity may be more sensitive to the specific measurement context than the relationship between environmental press and malevolent creativity, given the wider range of personality constructs and sample characteristics represented in the negative person literature.

MC indicator also significantly moderated effect sizes in the negative person category, with the largest effect observed for Hurting People. This pattern suggests that this dimension may most directly capture the core motivational component of malevolent creativity, namely the intentional desire to harm others. Prior work has shown that some maladaptive traits, including psychopathy, moral disengagement, and vengefulness, are associated with enjoyment of others' suffering (Perchtold-Stefan et al., 2023a). Consistent with this view, individuals who are more responsive to others' distress may be more motivated to generate a wider range of harmful ideas, increasing the likelihood of identifying effective means of causing harm (Perchtold-Stefan et al., 2022a).

### 4.2 Implications

The present meta-analysis extends prior work by using robust methods to examine heterogeneity in the links between antecedents and malevolent creativity (MC). These findings advance understanding of MC antecedents and also bear on broader questions about the conceptualization of MC. Negative press and negative person factors were both positively associated with MC, whereas positive person factors were negatively associated with MC. Individuals with positive traits, such as self-control and empathy, may reduce the maliciousness of generated ideas without necessarily diminishing novelty. Even under conflict, such individuals may be more likely to view prosocial responses as viable, leading to ideas that are relatively novel but less harmful (Baas et al., 2019).

Within the 4P framework, person-related antecedents showed greater between-study heterogeneity than press-related antecedents. This pattern likely reflects situational dependency rather than instability in the traits themselves. Consistent with the view of traits as relatively stable individual differences, person-related factors showed lower within-study variance, suggesting greater precision in estimating person effects. Overall, dispositional traits appeared more strongly linked to MC than situational press factors. Our findings may reflect the view that, compared with malevolent creativity triggered by external situational pressures, which may resemble reactive aggression, malevolent creativity rooted in dark personality traits may be more strongly associated with novel harmful ideation. In particular, aggression, indifference to others' suffering, and biased interpretations of the social world may predispose individuals to generate harmful ideas in a more proactive manner. Accordingly, fostering healthy personality development may be especially important. Support from schools, families, and broader social contexts may help children develop adaptive values and stronger moral standards, thereby reducing the likelihood that harmful tendencies are expressed in novel ways.

## 5 Conclusions

### 5.1 Summary and implications

This meta-analysis synthesized evidence from 55 studies and 362 effect sizes, reorganizing the MC literature within a 4P framework. Both negative person factors (*r* = 0.34) and negative press factors (*r* = 0.26) were significantly associated with malevolent creativity, with person-level antecedents showing a stronger overall effect. A formal statistical comparison confirmed that this difference was significant, *B* = 0.168, *SE* = 0.043, *z* = 3.938, *p* < 0.001, suggesting that stable dispositional characteristics exert a greater influence on malevolent creativity than situational environmental pressures. Positive person and press factors showed small negative associations with malevolent creativity, indicating that protective factors may attenuate harmful ideation without necessarily reducing creative potential.

Although the overall effects were modest, they were systematically shaped by MC measurement and MC indicator. The wide prediction intervals observed across all main effects, despite significant mean effects, highlight the substantial cross-study variability in these associations and underscore the context-dependent nature of malevolent creativity antecedents.

A key theoretical contribution of this meta-analysis is the proposal of a dual-pathway model integrating the proactive-reactive aggression distinction (Miller and Lynam, 2006) with the 4P framework. Person-level antecedents are proposed to operate through a proactive pathway, whereby stable dispositional traits such as Dark Triad characteristics and moral disengagement create a chronic readiness for harmful ideation that operates independently of situational context. Press-level antecedents are proposed to operate through a reactive pathway, whereby adverse environmental conditions activate negative emotions, particularly anger, that temporarily redirect creative cognition toward harmful ends. This framework provides a more coherent theoretical account of the existing literature than simple additive models and generates testable predictions for future research.

The alignment between measurement tools and pathway type further supports this framework. The MCBS, as a trait-level measure of everyday malevolent behavior frequency, appears to capture proactive malevolent creativity and shows stronger sensitivity to person-level antecedents. The MCT, as an acute experimental task simulating conflict and provocation, appears to capture reactive malevolent creativity and shows stronger sensitivity to press-level antecedents. This distinction has important implications for instrument selection in future primary studies.

In conducting this meta-analysis, we also observed substantial inconsistency in how MC is assessed, which likely limits interpretability and underscores the need for stronger methodological rigor in future research. More broadly, our findings suggest that the field should move beyond the generation of malevolent ideas and place greater emphasis on the processes through which such ideas are implemented.

These findings also carry important practical implications. The proactive nature of trait-driven malevolent creativity suggests that early interventions targeting personality development may be particularly effective, as support from schools, families, and broader social contexts may help individuals develop adaptive values, stronger moral standards, and greater empathic concern, thereby reducing the chronic readiness for harmful ideation (Gao et al., 2025a; Chen et al., 2025). For press-level interventions, reducing exposure to adverse environmental conditions and enhancing emotion regulation skills may help mitigate reactively triggered malevolent creativity (Perchtold-Stefan et al., 2024).

### 5.2 Limitations

Several limitations warrant note. First, our conclusions are constrained by the methodological quality of the included primary studies, particularly regarding sampling and data collection. Second, restricting inclusion to English-language publications may have introduced language bias; future work should incorporate non-English sources to ensure broader representativeness. Third, the predominance of East Asian samples, particularly from China, limits cultural generalizability. This highlights the need to examine how cultural contexts moderate the antecedents of malevolent creativity, as relationships observed here may not universally apply across Western society (Sternberg, 2009; Barth and Stadtmann, 2026). Fourth, the relatively smaller association between negative press and MC may reflect limitations of the task paradigms typically used in this literature. In many studies, the MCT requires participants to read a brief provocative scenario and generate responses within a restricted time window, which may not fully capture how malevolent ideas develop in everyday life. In real-world contexts, individuals high in self-control or premeditation may suppress immediate revenge impulses, engage in sustained angry rumination, and delay retaliation until they are better able to enact a planned response (Harris and Reiter-Palmon, 2015; Chester, 2024).

### 5.3 Future study

Future research must prioritize consistency in MC assessment, particularly regarding fluency scoring. Adhering to established criteria whereby a response is classified as malevolently creative only when meeting thresholds for both negative valence and novelty ensures fidelity to the construct's core dimensions (Lee and Dow, 2011). Beyond generation, scholarship must shift focus toward implementation, as potentially harmful ideas lack tangible impact until translated into behavior or products (Nguyen et al., 2024). Identifying the person and press factors that facilitate this transition constitutes a critical priority, particularly in organizational settings where emerging tools such as the Malevolent Innovative Behavior Scale (MIBS; Wang et al., 2025) offer promising avenues for measurement. Future research should also directly test the proposed dual-pathway model by examining whether person and press factors interact in predicting malevolent creativity, and whether MCBS and MCT show differential sensitivity to proactive vs. reactive antecedents as hypothesized.

## Data Availability

The original contributions presented in the study are included in the article/supplementary material, further inquiries can be directed to the corresponding author/s.

## References

[B1] Abdulla Alabbasi A. M. Thompson T. L. Runco M. A. Alansari L. A. Ayoub A. E. A. (2025). Gender differences in creative potential: a meta-analysis of mean differences and variability. Psychol. Aesthet. Creat. 19:87. doi: 10.1037/aca0000506

[B2] Assink M. Van Der Put C. E. Hoeve M. De Vries S. L. A. Stams G. J. J. M. Oort F. J. (2015). Risk factors for persistent delinquent behavior among juveniles: a meta-analytic review. Clin. Psychol. Rev. 42, 47–61. doi: 10.1016/j.cpr.2015.08.002 26301752

[B3] Assink M. Wibbelink C. J. M. (2016). Fitting three-level meta-analytic models in r: a step-by-step tutorial. TQMP 12, 154–174. doi: 10.20982/tqmp.12.3.p154

[B4] Baas M. Roskes M. Koch S. Cheng Y. De Dreu C. K. W. (2019). Why social threat motivates malevolent creativity. Pers. Soc. Psychol. Bull. 45, 1590–1602. doi: 10.1177/0146167219838551 30931827

[B5] Bandura A. (2017). “Moral disengagement in the perpetration of inhumanities,” in Recent developments in criminological theory, eds. F. T. Cullen, R. Agnew, and P. Wilcox (London: Routledge), 135–152. doi: 10.4324/9781315089089-12

[B6] Barth P. Stadtmann G. (2026). Creativity in the west and the east: a meta-analysis of cross-cultural differences. Creat. Res. J. 38, 1–47. doi: 10.1080/10400419.2024.2369442

[B7] Batey M. Hughes D. J. Mosley A. Owens C. E. Furnham A. (2022). Psychopathy and openness-to-experience as predictors of malevolent and benevolent creativity. Pers. Individ. Differ. 196:111715. doi: 10.1016/j.paid.2022.111715

[B8] Berkowitz L. (1990). On the formation and regulation of anger and aggression: a cognitive-neoassociationistic analysis. Am. Psychol. 45:494. doi: 10.1037/0003-066X.45.4.494 2186678

[B9] Buss A. H. Finn S. E. (1987). Classification of personality traits. J. Pers. Soc. Psychol. 52, 432–444. doi: 10.1037/0022-3514.52.2.432

[B10] Card N. A. (2015). Applied Meta-Analysis for Social Science Research. New York, NY: Guilford Publications.

[B11] Card N. A. Stucky B. D. Sawalani G. M. Little T. D. (2008). Direct and indirect aggression during childhood and adolescence: a meta-analytic review of gender differences, intercorrelations, and relations to maladjustment. Child Dev. 79, 1185–1229. doi: 10.1111/j.1467-8624.2008.01184.x 18826521

[B12] Ceballos N. A. Watt T. T. (2023). The influence of adverse childhood experiences on malevolent creativity in young adulthood. Behav. Sci. 13:961. doi: 10.3390/bs13120961 38131817 PMC10740602

[B13] Chandler J. Cumpston M. Li T. Page M. J. Welch V. (2019). Cochrane Handbook for Systematic Reviews of Interventions. Hoboken: Wiley.

[B14] Chen F. Xiao Y. Wang Y. Huang Y. Yang W. Cao G. (2025). The link between family support and malevolent creativity: the mediating roles of reflective rumination and brooding rumination. Psychol. Rep. 4:332941251363462 4:332941251363462. doi: 10.1177/00332941251363462 40757744

[B15] Chester D. S. (2024). Aggression as successful self-control. Soc. Personal. Psychol. Compass 18:e12832. doi: 10.1111/spc3.12832

[B16] Cheung M. W.-L. (2014). Modeling dependent effect sizes with three-level meta-analyses: a structural equation modeling approach. Psychol. Methods 19:211. doi: 10.1037/a0032968 23834422

[B17] Crick N. R. Dodge K. A. (1996). Social information-processing mechanisms in reactive and proactive aggression. Child Dev. 67, 993–1002. doi: 10.2307/1131875 8706540

[B18] Cropley D. H. Cropley A. J. Kaufman J. C. Runco M. A. Eds. (2010). Malevolent innovation: opposing the dark side of creativity, 33–56. Cambridge: Cambridge University Press.

[B19] Cropley D. H. Kaufman J. C. Cropley A. J. (2008). Malevolent creativity: a functional model of creativity in terrorism and crime. Creativity Res. J. 20, 105–115. doi: 10.1080/10400410802059424

[B20] Cui X. Zhang X. Zhang H. (2024). The impact of parenting style on malevolent creativity based on Chinese university students: a latent profile analysis. Front. Psychol. 15:1363778. doi: 10.3389/fpsyg.2024.1363778 38988383 PMC11233728

[B21] da Costa S. Páez D. Sánchez F. Garaigordobil M. Gondim S. (2015). Personal factors of creativity: a second order meta-analysis. Rev. Psicol. Trab. Organ. 31, 165–173. doi: 10.1016/j.rpto.2015.06.002

[B22] ^*^ d'Amato A. L. Theobald E. Scott M. N. Elson J. S. Hunter S. T. (2025). Examining similarity-attraction principle and intergroup conflict on malevolent creativity ideation. J. Creat. Behav. 59:e70027. doi: 10.1002/jocb.70027

[B23] ^*^De Farias E. S. Bonfá-Araujo B. Nakano T. D. C. Campos C. R. (2025). Malevolent creativity behavior scale-Brazilian Portuguese: cross-cultural adaptation and psychometric properties. J. Creat. Behav. 59:e70009. doi: 10.1002/jocb.70009

[B24] ^*^Du X. Zhao Y. Zhang K. (2024). The influence of group categorization and common ingroup identity on malevolent creativity, benevolent creativity, and neutral creativity. Think. Skills Creat. 54:101686. doi: 10.1016/j.tsc.2024.101686

[B25] Dumas D. G. Strickland A. L. (2018). From book to bludgeon: a closer look at unsolicited malevolent responses on the alternate uses task. Creat. Res. J. 30, 439–450. doi: 10.1080/10400419.2018.1535790

[B26] Endler N. S. (2000). The interface between personality and cognition. Eur. J. Pers. 14, 377–389. doi: 10.1002/1099-0984(200009/10)14:5<377::AID-PER390>3.0.CO;2-2

[B27] Fernández-Castilla B. Declercq L. Jamshidi L. Beretvas S. N. Onghena P. Van Den Noortgate W. (2021). Detecting selection bias in meta-analyses with multiple outcomes: a simulation study. J. Exp. Educ. 89, 125–144. doi: 10.1080/00220973.2019.1582470 32808180

[B28] ^*^Fousiani K. Xu S. Van Prooijen J. (2025). Leaders' power construal influences malevolent creativity: the mediating role of organizational conspiracy beliefs. J. Occup. Organ. Psychol. 98:e70005. doi: 10.1111/joop.70005

[B29] ^*^Fu H. Zhang Z. (2024). The relationship between honesty-humility and malevolent creativity: sequential mediation models with prosocial moral emotional traits and prosocial tendencies. Curr. Psychol. 43, 7424–7436. doi: 10.1007/s12144-023-04941-2

[B30] Gao Z. Cheng L. Li J. Chen Q. Hao N. (2022a). The dark side of creativity: neural correlates of malevolent creative idea generation. Neuropsychologia 167:108164. doi: 10.1016/j.neuropsychologia.2022.108164 35085597

[B31] Gao Z. Liu X. Gao M. Hao N. (2025a). Neural correlates underlying creative ideation associated with malevolent or benevolent intentions. Cereb. Cortex 35:bhaf010. doi: 10.1093/cercor/bhaf010 39917814

[B32] ^*^Gao Z. Qiao X. Lu K. Wang X. Hao N. (2025b). Dynamic amplitude of low-frequency fluctuation links dark personalities to malevolent creative behavior. Brain Cogn. 183:106245. doi: 10.1016/j.bandc.2024.106245 39657373

[B33] ^*^Gao Z. Qiao X. Xu X. Hao N. (2022b). Darkness within: the internal mechanism between dark triad and malevolent creativity. J. Intell. 10:119. doi: 10.3390/jintelligence10040119 36547506 PMC9784164

[B34] Ge Y. Wu L. Pan B. Liu Y. Gao Z. Qiao X. . (2026). The influence of social exclusion on bystanders' retaliatory malevolent creativity. Think. Skills Creat. 59:101964. doi: 10.1016/j.tsc.2025.101964

[B35] Geng Y. Shi Y. Hu W. Jin W. Zhang Y. Zhan T. (2024). Fight injustice with darkness: the effect of early life adversity on malevolent creativity behavior. J. Creat. Behav. 58:279. doi: 10.1002/jocb.648

[B36] Gfrörer T. Stoll G. Rieger S. Trautwein U. Nagengast B. (2022). The development of vocational interests in early adolescence: stability, change, and state-trait components. Eur J PersEur. J. Pers. 36, 942–964. doi: 10.1177/08902070211035630

[B37] Gutworth M. B. Cushenbery L. Hunter S. T. (2018). Creativity for deliberate harm: malevolent creativity and social information processing theory. J. Creat. Behav. 52, 305–322. doi: 10.1002/jocb.155

[B38] Haase J. Hanel P. H. P. Gronau N. (2025). Creativity enhancement methods for adults: a meta-analysis. Psychol. Aesthet. Creat. 19, 708–736. doi: 10.1037/aca0000557

[B39] ^*^Hameed I. (2025). Crafting harm: How Machiavellianism, competitive worldview, and supervisor bottom-line mentality fuel malevolent creativity? *J. Econ. Adm. Sci*. doi: 10.1108/JEAS-10-2024-0408

[B40] ^*^Hao N. Qiao X. Cheng R. Lu K. Tang M. Runco M. A. (2020). Approach motivational orientation enhances malevolent creativity. Acta Psychol. 203:10. doi: 10.1016/j.actpsy.2019.102985 31863973

[B41] Hao N. Tang M. Yang J. Wang Q. Runco M. A. (2016). A new tool to measure malevolent creativity: the malevolent creativity behavior scale. Front. Psychol. 7:7. doi: 10.3389/fpsyg.2016.00682 27242596 PMC4870273

[B42] Harris D. J. Reiter-Palmon R. (2015). Fast and furious: the influence of implicit aggression, premeditation, and provoking situations on malevolent creativity. Psychol. Aesthet. Creat. Arts 9, 54–64. doi: 10.1037/a0038499

[B43] Harris D. J. Reiter-Palmon R. Kaufman J. C. (2013). The effect of emotional intelligence and task type on malevolent creativity. Psychol. Aesthet. Creat. Arts 7, 237–244. doi: 10.1037/a0032139

[B44] Huang W. Kong L. Wu Y. Liu L. Huang S. Tu M. . (2026). School bullying predicts malevolent creativity in middle school students through anger and hostile attribution bias. Sci. Rep. 16:5259. doi: 10.1038/s41598-026-36211-z 41535571 PMC12881546

[B45] Jia X. Wang Q. Lin L. (2020). The relationship between childhood neglect and malevolent creativity: the mediating effect of the dark triad personality. Front. Psychol. 11:613695. doi: 10.3389/fpsyg.2020.613695 33391134 PMC7772220

[B46] ^*^Kapoor H. Kaufman J. C. (2022). Unbound: the relationship among creativity, moral foundations, and dark personality. J. Creat. Behav. 56, 182–193. doi: 10.1002/jocb.523

[B47] ^*^Khorakian A. Hemsworth D. Jahangir M. Maharati Y. Bagherpour E. S. Muterera J. (2020). The effects of religious orientations on malevolent creativity: role of positive emotions and spiritual intelligence. Creat. Res. J. 32, 421–430. doi: 10.1080/10400419.2020.1818491

[B48] Kim J. Vaulont M. J. Zhang Z. Byron K. (2024). Looking inside the black box of gender differences in creativity: a dual-process model and meta-analysis. J. Appl. Psychol. 109, 1861–1900. doi: 10.1037/apl0001205 39073386

[B49] Kmet L. M. Cook L. S. Lee R. C. (2004). Standard Quality Assessment Criteria for Evaluating Primary Research Papers from a Variety of Fields. Edmonton, AB: Alberta Heritage Foundation for Medical Research.

[B50] Lee S. A. Dow G. T. (2011). Malevolent creativity: does personality influence malicious divergent thinking? Creativity Res. J. 23, 73–82. doi: 10.1080/10400419.2011.571179

[B51] Li L. Hou Y. Ouyang L. (2025a). The impact of social exclusion on malevolent creativity among Chinese university students: the roles of empathy, moral disengagement, and teacher support. Sci. Rep. 15:38368. doi: 10.1038/s41598-025-22233-6 41184360 PMC12583578

[B52] Li T. Han J. Wan Z. Pan X. Li R. Yao C. (2025b). The relationship between bullying victimization and malevolent creativity in Chinese middle school students: a moderated chain mediation model. Behav. Sci. 15:1386. doi: 10.3390/bs15101386 41153176 PMC12561838

[B53] Li W. Zhang L. Qin Z. Chen J. Liu C. (2022). Childhood trauma and malevolent creativity in Chinese college students: moderated mediation by psychological resilience and aggression. J. Intell. 10:97. doi: 10.3390/jintelligence10040097 36412777 PMC9680388

[B54] ^*^Li W. Zhou Y. (2025). Smartphone addiction and malevolent creativity—the mediating role of psychological capital and the moderating role of self-concept clarity. Front. Psychiatry 16:1574738. doi: 10.3389/fpsyt.2025.1574738 40357510 PMC12066462

[B55] ^*^Li Y. Gong Z. Xie P. (2025c). Cruel creativity delight in malevolence: development and testing of the malevolent sadistic creativity behavior tendencies scale. J. Creat. Behav. 59:e70049. doi: 10.1002/jocb.70049

[B56] ^*^Malik O. F. Shahzad A. Waheed A. Yousaf Z. (2020). Abusive supervision as a trigger of malevolent creativity: do the light triad traits matter? Leadersh. Organ. Dev. J. 41, 1119–1137. doi: 10.1108/LODJ-09-2019-0386

[B57] Miller J. D. Lynam D. R. (2006). Reactive and proactive aggression: similarities and differences. Pers. Individ. Dif. 41, 1469–1480. doi: 10.1016/j.paid.2006.06.004

[B58] Nguyen T. L. d'Amato A. L. Miller S. R. Hunter S. T. (2025). Malevolent creativity as parochial altruism? examining the intergroup bases of new and harmful ideas. Creat. Res. J. 37:104. doi: 10.1080/10400419.2023.2255011

[B59] Nguyen T. L. Walters K. N. d'Amato A. L. Miller S. R. Hunter S. T. (2024). Target personification influences the positive emotional link between generating and implementing malevolently creative ideas. Creativity Res. J. 36, 42–57. doi: 10.1080/10400419.2022.2089820

[B60] Ouzzani M. Hammady H. Fedorowicz Z. Elmagarmid A. (2016). Rayyan—a web and mobile app for systematic reviews. Syst. Rev. 5:210. doi: 10.1186/s13643-016-0384-4 27919275 PMC5139140

[B61] Perchtold-Stefan C. M. Fink A. Rominger C. Papousek I. (2021a). Creative, antagonistic, and angry? Exploring the roots of malevolent creativity with a real-world idea generation task. J. Creative Behav. 55, 710–722. doi: 10.1002/jocb.484 34690361 PMC8518065

[B62] ^*^Perchtold-Stefan C. M. Fink A. Rominger C. Papousek I. (2021b). Failure to reappraise: malevolent creativity is linked to revenge ideation and impaired reappraisal inventiveness in the face of stressful, anger-eliciting events. Anxiety Stress Coping 34, 437–449. doi: 10.1080/10615806.2021.1918682 33899626 PMC8367047

[B63] Perchtold-Stefan C. M. Fink A. Rominger C. Papousek I. (2024). Social exclusion increases antisocial tendencies: evidence from retaliatory ideation in a malevolent creativity task. Psychol. Aesthet. Creat. Arts 18, 1014–1025. doi: 10.1037/aca0000500

[B64] Perchtold-Stefan C. M. Fink A. Rominger C. Szabó E. Papousek I. (2022a). Enjoying others' distress and indifferent to threat? Changes in prefrontal-posterior coupling during social-emotional processing are linked to malevolent creativity. Brain Cogn. 163:105913. doi: 10.1016/j.bandc.2022.105913 36087513

[B65] Perchtold-Stefan C. M. Rominger C. Fink A. (2023a). Depressive symptoms are positively linked to malevolent creativity: a novel perspective on the maladaptive nature of revenge ideation. J. Creative Behav. 57, 319–330. doi: 10.1002/jocb.580

[B66] Perchtold-Stefan C. M. Rominger C. Papousek I. Fink A. (2022b). Antisocial schizotypy is linked to malevolent creativity. Creativity Res. J. 34, 355–367. doi: 10.1080/10400419.2021.2012633

[B67] ^*^Perchtold-Stefan C. M. Rominger C. Papousek I. Fink A. (2023b). Women and men have a similar potential for malevolent creativity – but their underlying brain mechanisms are different. Brain Res. 1801:148201. doi: 10.1016/j.brainres.2022.148201 36521515

[B68] Pigott T. D. Polanin J. R. (2020). Methodological guidance paper: high-quality meta-analysis in a systematic review. Rev. Educ. Res. 90, 24–46. doi: 10.3102/0034654319877153

[B69] Quetelet A. (1831). Research on the Propensity to Crime of Different Ages. Translt by F. Sawyer & T. Sylvester. Brussels: Hayez.

[B70] Rhodes M. (1961). *An analysis of creativity*. The Phi delta kappan 42, 305–310. Available online at: https://www.jstor.org/stable/20342603 (Accessed March 14, 2026).

[B71] Riddell H. Sedikides C. Sivaramakrishnan H. Wan P. Maltagliati S. Jackson B. . (2025). A meta-analytic review and conceptual model of the antecedents and outcomes of goal adjustment in response to striving difficulties. Nat. Hum. Behav. doi: 10.1038/s41562-025-02312-4 41233533 PMC12932095

[B72] Said-Metwaly S. Taylor C. L. Camarda A. Barbot B. (2022). Divergent thinking and creative achievement—how strong is the link? An updated meta-analysis. Psychol. Aesthet. Creat. Arts. 18:869.

[B73] ^*^Segatta C. Pozzoli T. Agnoli S. (2025). Moral disengagement as a main component of malevolent creative potential. Creativity. Theories Res. Appl. 12, 1–20. doi: 10.2478/ctra-2025-0001

[B74] Shi Z. Zhou Z. Tian L. Zhu Y. Liu C. Xu L. (2023). What causes malevolent creative people to engage in malevolent behaviors? Mediating role of moral disengagement and moderating effects of conscience. Think. Skills Creat. 49:101329. doi: 10.1016/j.tsc.2023.101329

[B75] Silva D. Junça-Silva A. Pinheiro P. (2025). Folie à deux? How Mavericks shape the relationship between the dark triad and negative deviant behaviours through malevolent creativity. Pers. Individ. Differ. 233:112877. doi: 10.1016/j.paid.2024.112877

[B76] Simonton D. K. (1994). Greatness: Who Makes History and why. New York, NY: Guilford Press.

[B77] Sternberg R. J. (2009). “Domain-Generality Vs. Domain-Specificity of Creativity,” in Milieus of Creativity, eds. P. Meusburger, J. Funke, and E. Wunder (Dordrecht: Springer Netherlands), 25–38. doi: 10.1007/978-1-4020-9877-2_3

[B78] Sternberg R. J. Karami S. (2022). An 8P theoretical framework for understanding creativity and theories of creativity. J. Creat. Behav. 56, 55–78. doi: 10.1002/jocb.516

[B79] Storme M. Celik P. Myszkowski N. (2021). Creativity and unethicality: a systematic review and meta-analysis. Psychol. Aesthet. Creat. Arts 15, 664–672. doi: 10.1037/aca0000332

[B80] Szabó E. Körmendi A. Kurucz G. Cropley D. Olajos T. Pataky N. (2022). Personality traits as predictors of malevolent creative ideation in offenders. Behav. Sci. 12:242. doi: 10.3390/bs12070242 35877312 PMC9311653

[B81] ^*^Tong D. Shi Y. Gu X. Lu P. (2024). Bullying victimization and malevolent creativity in rural adolescents: the longitudinal mediational role of hostile attribution. Cyberpsychol. Behav. Soc. Netw. 27:0499. doi: 10.1089/cyber.2023.0499 38511278

[B82] Triandis H. C. (1989). The self and social behavior in differing cultural contexts. Psychol. Rev. 96:506. doi: 10.1037/0033-295X.96.3.506

[B83] Viechtbauer W. (2010). Conducting meta-analyses in *R* with the metafor Package. J. Stat. Soft. 36:i03. doi: 10.18637/jss.v036.i03

[B84] Viechtbauer W. Cheung M. W.-L. (2010). Outlier and influence diagnostics for meta-analysis. Res. Synth. Methods 1, 112–125. doi: 10.1002/jrsm.11 26061377

[B85] ^*^Wang Q. (2023). The effect of parenting practices on creativity: mediating role of psychological resilience. Psychol. Res. Behav. Manag. 16, 4501–4514. doi: 10.2147/PRBM.S436370 37936972 PMC10627049

[B86] Wang Y. Rietzschel E. F. Wisse B. (2025). Malevolent innovation: a synthesized theoretical model and a measurement tool. Psychol. Aesthet. Creat. Arts.doi: 10.1037/aca0000782

[B87] ^*^Wang Y. Zhang K. Xu F. Zong Y. Chen L. Li W. (2024). The effect of justice sensitivity on malevolent creativity: the mediating role of anger and the moderating role of emotion regulation strategies. BMC Psychol. 12:265. doi: 10.1186/s40359-024-01759-w 38741161 PMC11092024

[B88] ^*^Wu J. Ma F. Liu J. Jiao L. (2025). The effects of different types of social exclusion on malevolent creativity: the role of self-construal. Think. Skills Creat. 57:101837. doi: 10.1016/j.tsc.2025.101837

[B89] Wu J. Rao X. Liu J. You S. Cao Y. Jiao L. (2024). The Impact of Social Exclusion on Malevolent Creativity: The Mediating Role of Prosocial Motivation. Washington, DC: Psychology of Aesthetics, Creativity, and the Arts.

[B90] Xu X. Xia M. Pang W. (2023). Do all roads lead to Rome? Authenticity, openness to experience, and risk-taking relate to general and malevolent creativity differently. Curr. Psychol. 42, 13424–13431. doi: 10.1007/s12144-021-02567-w

[B91] Xu X. Zhao J. Xia M. Pang W. (2021). I can, but i won't: authentic people generate more malevolently creative ideas, but are less likely to implement them in daily life. Pers. Individ. Dif. 170:5. doi: 10.1016/j.paid.2020.110431

[B92] ^*^Yang B. Li H. (2026). How multicultural experiences influence malevolent creativity. Psychol. Rep. 129, 519–539. doi: 10.1177/00332941241233208 38334333

[B93] ^*^Yu L. Qiao X. Hao N. (2024). Intergroup threat stimulates malevolent creative idea generation. Motiv. Emot. 48:531. doi: 10.1007/s11031-024-10070-5

[B94] ^*^Zhang J. Lu J. Ge J. Li S. Liang X. (2025). Managing malice in negative environments: the mediating effect of coping styles on the relationship between negative sense of place and malevolent creativity among Chinese high school students. BMC Psychol. 13:35. doi: 10.1186/s40359-024-02333-0 39806442 PMC11731381

[B95] Zhang W. Liang Q. Qiao X. Hao N. (2024). ‘brings malice: malevolent creativity is modulated by perceived unfairness of others. Think. Skills Creat. 53:101586. doi: 10.1016/j.tsc.2024.101586

[B96] Zhao J. Xu X. Pang W. (2022a). When do creative people engage in malevolent behaviors? The moderating role of moral reasoning. Pers. Individ. Differ. 186:111386. doi: 10.1016/j.paid.2021.111386

[B97] Zhao M. Zhang K. Du X. (2022b). The effect of infectious disease threat on malevolent creativity. J. Intell. 10:111. doi: 10.3390/jintelligence10040111 36412791 PMC9680440

[B98] Zhou J. Zhao B. Li Y. (2024). A network meta-analysis of factors influencing malevolent creativity. Soc. Behav. Pers. 52, 1–18. doi: 10.2224/sbp.12940

[B99] ^*^Zivi P. Giancola M. Nori R. Piccardi L. D'Amico S. Palmiero M. (2025). Field dependent-independent cognitive style as a predictor of malevolent creativity: a multifaceted approach. Front. Psychol. 16:1502823. doi: 10.3389/fpsyg.2025.1502823 40672842 PMC12263601

